# An Easy Method of Making a Gold Crown for Anterior Teeth

**Published:** 1904-06

**Authors:** J. M. King

**Affiliations:** Brookside, Ala.


### AN EASY METHOD OF MAKING A GOLD CROWN FOR ANTERIOR TEETH.

By J. M. KING, D. D. S., Brookside, Ala.

The most economical, quickest and best way to make anterior gold crowns is to grind the square end of the dentist's anvil in a shape for contouring and then give a high polish. Obtain a small hammer best suited for your use, take measurement of root and make band of 30-gauge gold, fit on model and contour next to gum with hammer and anvil and crescent contour flush. Trim band so it will go under free margin of gum, contour band, trim plaster tooth and trim band lingually so as to get an alignment. Then trim bite from band, and solder a piece of gold over this which makes the work complete. A little pains will enable any one to make a crown which has extra good shape and the thickness of the gold will allow for a good finish. Use tripoli for polishing and then a chamois polishing wheel with rouge thoroughly incorporated in it. This rouge gives it a beautiful finish. A little practice will train the operator to make the so-called hand-made crowns which look well—

though I am not a strong advocate of anterior gold crowns. Every practitioner has some to make sometime in his life and to be able to do it at home is of great advantage.—*Summary.*

